# Quantification of feeding intensity and feeding control of largemouth bass based on water surface vibration characteristics

**DOI:** 10.3389/frai.2026.1656290

**Published:** 2026-03-13

**Authors:** Yufei Zhang, Andong Liu, Yulei Zhang, Qi Ni, Haigen Zhang, Hongqiao Song, Yong Wang, Xiaoyan Cheng

**Affiliations:** 1 Fishery Machinery and Instruction Research Institute, Shanghai, China; 2 School of Navigation and Naval Architecture, Dalian Ocean University, Dalian, China; 3 Laiyang Fishery Technology Extension Station, Yantai, Shandong, China

**Keywords:** aquaculture, fish appetite quantification, fish feeding model, fluctuations in fish feeding, online intelligent feeding

## Abstract

In response to the demand for precise feeding in high-density aquaculture, this study established a dynamic prediction model for fish feeding intensity by integrating vibration signal quantification and deep learning. Through multidimensional experiments (fish size: 50–300 g; stocking density: 20–60 fish/group; feeding speed: 1-3 g/s; feed particle size: 2#4#6#), we quantified the three-axis displacement signals of *Micropterus salmoides* during feeding. Results demonstrated significant effects of all parameters on water surface fluctuations (*p* < 0.05). Vibration displacement exhibited linear relationships with fish size and density. The 300 g group showed 109.7% higher peak amplitude than the 50 g group, while the 60-fish density group exceeded the 20-fish group by 141.9%. Optimal palatability (4#) reduced fluctuation frequency by 42%. A predictive model for feeding vibration patterns was developed, incorporating fish size (S), density (D), feeding speed (V), feed particle size (*Φ*), real-time triaxial vibration sum, and time series (t) as inputs to predict the summed vibration displacement at t + 5 s, which serves as a quantitative proxy for feeding intensity. The Long Short-Term Memory (LSTM) model accurately captured fish feeding dynamics (RMSE = 69.43 μm, MAE = 48.00 μm, R^2^ = 0.883). In comparative analysis, the LSTM outperformed Gated Recurrent Unit (GRU) and Transformer models in forecasting accuracy. Deployed on an embedded system (Orange Pi AiPRO), closed-loop tests demonstrated superior performance: residual feed rates were ≤ 0.8% across all trials, outperforming optical flow (2.69% residuals) and graph neural network (6.58% residuals) methods. The space complexity of the vibration-LSTM approach was only 6.4–31.8% of GCN-based approaches, enabling cost-effective (<$200) real-time control.

## 1 Introduction

In factory-based largemouth bass farming, feed costs account for more than 60% of the total farming costs, and whether feeding is reasonable is directly related to the profit margin of aquatic products (Zhou et al., 2018). Traditional feeding methods usually rely on the experience and intuition of skilled workers. However, with the aging of the population, the labor shortage problem is becoming more and more serious, and manual feeding alone can no longer meet the needs of industrial scale (Zheng and Zheng, 2025). Although various automatic feeders have appeared on the market, conventional automatic feeder technology cannot achieve autonomous decision-making, and its promotion and application are greatly restricted. Therefore, intelligent feeding technology with autonomous decision-making is the future development direction of fish feeding methods (Zhu et al., 2022).

Recent research has focused on estimating fish feeding intensity by analyzing behavioral signals through machine vision, acoustics, and water surface fluctuations (Wu et al., 2022; Biazi and Marques, 2023; Føre et al., 2024). Vision-based methods, such as optical flow and deep learning models, classify feeding activity into discrete states (e.g., none, weak, medium, and strong) with high accuracy (>90%) (Xu et al., 2022; Hu et al., 2025). Advanced techniques like 3D convolutional neural networks and Graph Convolutional Networks (GCNs) have further improved performance by modeling spatiotemporal dynamics of fish schools (Ubina et al., 2021).

Acoustic methods analyze sounds generated during feeding—such as chewing, swallowing, and bait impacts—using underwater hydrophones. By leveraging time-frequency representations (e.g., Mel spectrograms, STFT) and feature fusion strategies, these approaches achieve high classification accuracy (>96%) (Qi et al., 2023; Du et al., 2023a,b). Transformer-based models, such as the Audio Spectrum Swin Transformer, support rapid inference and real-time monitoring of feeding intensity (Zeng et al., 2023). However, acoustic methods are susceptible to ambient noise from operational equipment (e.g., pumps, aerators), which can mask biologically relevant signals. To address this, multimodal fusion frameworks that combine audio, video, and sonar data have been proposed, achieving robustness in challenging conditions (Du et al., 2024).

Vibration-based methods, which capture water surface fluctuations via accelerometers mounted on floating platforms, offer a complementary approach. Xiang (2022) developed an adaptive feeding algorithm based on three-axis accelerometer feedback and proposed a quantitative index of feeding intensity. Pan et al. (2023) used a six-axis accelerometer to identify distinct feeding stages in crucian carp, leading to an automatic control method that improved feed utilization by 7.66%.

Despite these advances, current appetite assessment often relies on subjective visual evaluation, producing only qualitative labels without quantifiable metrics. Furthermore, systematic relationships between water surface fluctuations and key culture parameters—such as fish size, stocking density, feeding rate, and feed particle size-remain unestablished for species like largemouth bass. Crucially, existing studies classify appetite into discrete states but do not support the continuous, quantitative prediction of feeding intensity essential for dynamic, proportional feed control.

To bridge this gap, we propose a predictive model based on Long Short-Term Memory (LSTM) networks. LSTMs, a type of recurrent neural network (RNN), excel at capturing long-range temporal dependencies in sequential data (Hochreiter and Schmidhuber, 1997). Feeding behavior in largemouth bass is a continuous dynamic process where current surface vibrations are influenced by prior states. LSTM’s gating mechanisms mitigate the vanishing gradient problem common in standard RNNs, making the network particularly suitable for modeling such temporal sequences and predicting future fluctuations. Therefore, we adopt an LSTM architecture to model the complex, time-dependent relationships among multi-dimensional inputs (fish size, density, feeding rate, and feed size), real-time triaxial vibration signals, and future feeding intensity. The model’s output is the predicted future vibration displacement. We then utilize this displacement value as a continuous, quantitative proxy for feeding intensity, which forms the basis for dynamic feed control.

## 2 Materials and methods

### 2.1 Ethical statement

All research on animal participation in this article has passed the welfare and ethical review of experimental animals by FMIRI (FMIRI-AWE-2024-001). It should be noted that our experiments were conducted in an industrial recirculating water system (RAS) given its indispensable role in aquaculture and its rapid growth trend.

### 2.2 Experimental system

The recirculating aquaculture system (RAS) comprised six 1-m^3^ circular rearing tanks (water volume: 600 L; radius: 75 cm, water depth: 40 cm), each equipped with a screw-type feeder (feeding speed: 0–5 g/s). Water quality was rigorously controlled: temperature 26–30 °C, dissolved oxygen ≥5 mg/L (maintained at 5.5 ± 0.5 mg/L during acclimation/experiment), pH 7.2–8.5 (maintained at 7.2 ± 0.5 during acclimation/experiment), nitrate ≤0.5 mg/L, and total ammonia nitrogen (TAN) ≤ 0.8 mg/L.

**Figure 1:**
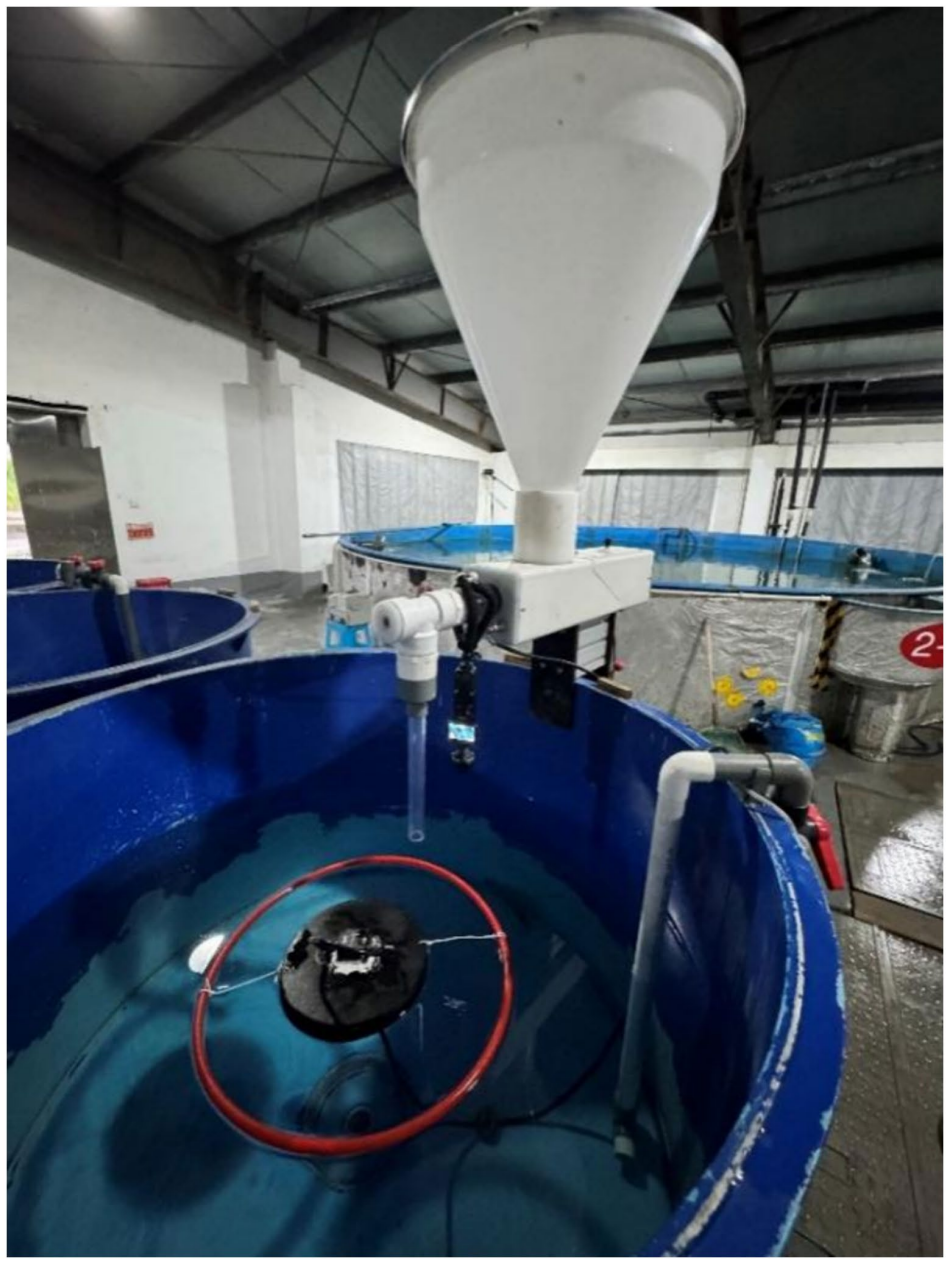
Experimental system device.

Vibration data acquisition employed a WT-VB01-485 triaxial sensor (47 × 38 × 15 mm) embedded within a 60° hardened EVA float (Ø 180 × 30 mm), with electronic components waterproofed using conformal coating. This sensor assembly was secured 15–20 cm downstream of the feeder outlet via an 80 g silicone-weighted data line and surrounded by a Ø 60 cm float ring to confine floating feed dispersion. Video monitoring utilized a camera (120 fps, 1920 × 1,080 resolution) mounted 40 cm above the water surface on an adjustable bracket. Real-time observational control was facilitated through Wi-Fi connectivity to a mobile device (Figure 1).

### 2.3 Experimental design

Juvenile largemouth bass (*Micropterus salmoides*) were grouped by size: small (50 g ± 4 g), medium (150 g ± 7 g), and large (300 g ± 11 g). Fish were stocked at densities of 20–60 per tank and fed once daily (16:00) with Tongwei California bass expanded feed (Specifications 2#, 4#, 6#; Crude protein ≥46.0%, Crude fat ≥6.0%). The feed input rate was set to 1, 2, or 3 g/s as an experimental variable. To ensure statistical robustness, each experimental condition (e.g., each fish size or density level) was replicated across two independent rearing tanks, with three repeated feeding trials conducted per tank. The detailed experimental design is summarized in Table 1.

**Table 1:** Experimental design and parameters for the feeding trials.

Experiment name	Fish weight (g)	Number of fish per tank (n)	Feed input rate (g/s)	Feed particle size
Size experiment	50, 150, 300	30	1	4#
Density experiment	50	20, 40, 60	1	4#
Feed input rate experiment	300	30	1, 2, 3	4#
Feed particle size experiment	300	30	1	2#, 4#, 6#

### 2.4 Overview of the proposed approach

**Figure 2:**
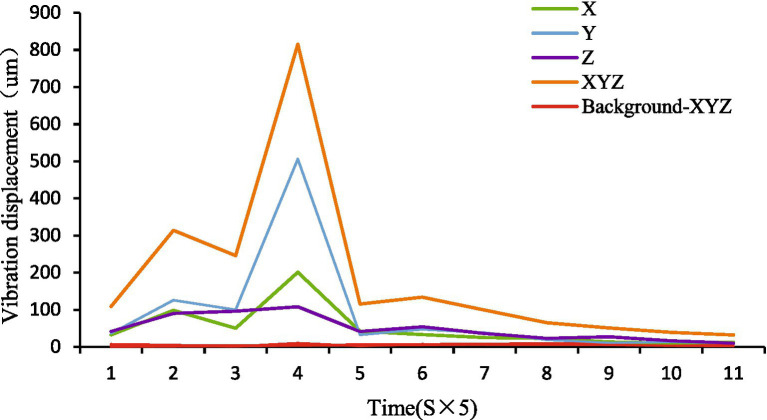
Water surface fluctuation parameter extraction.

To address the demand for precise feeding control in high-density aquaculture, this study established a dynamic prediction model for fish feeding intensity by integrating vibration signal quantification and deep learning. The research workflow comprised three key stages (Figure 2):

Analysis of Feeding Fluctuation Patterns: Through synchronous video verification, we analyzed the vibration characteristics of largemouth bass during feeding under variations in key parameters: fish size (S), stocking density (D), feeding rate (V), and feed particle size (*Φ*).

Prediction Modeling of Water Surface Fluctuations: A predictive model was developed using the multi-dimensional experimental data. The input variables included time (t), the static parameters (S, D, V, *Φ*), and the real-time triaxial vibration displacement. The output variable was the summed vibration displacement over the next 5 s (prediction horizon *N* = 5), representing a quantitative proxy for the predicted feeding intensity. The *Z*-score normalization method was applied to eliminate dimensional differences and enhance model convergence. The modeling architecture utilized a Long Short-Term Memory (LSTM) network. All experimental runs (408 trials in total) were pooled into a unified dataset. Each data instance consisted of a 5-s window (sampled at 10 Hz) of the summed triaxial vibration displacement, paired with the corresponding static parameters (S, D, V, Φ). This structured dataset was then randomly split into training (80%) and validation (20%) sets for model development.

Algorithm Deployment and Feeding Control Testing: The trained model was compressed and deployed on an embedded system (Orange Pi AiPRO) for real-time prediction (with a delay ≤ 50 ms). The feeding system adjusted the feeding rate (ΔV) based on the real-time feedback of the predicted feeding intensity, enabling anticipatory control, including early feeding cessation. The performance of this algorithm was rigorously compared against optical flow and graph neural network (GCN) methods to evaluate its efficacy.

### 2.5 Data preprocessing and feature engineering

The triaxial displacement signals (*X*, *Y*, *Z*) were measured using the WT-VB01-485 sensor. The X and Y axes represent horizontal displacements on the water surface plane, capturing lateral wave movements, while the Z axis corresponds to vertical displacement, capturing heave motion. This configuration allows comprehensive quantification of surface agitation dynamics. Figure 2 compares feeding vibrations versus environmental noise (aeration bubbles/water flow), confirming that our filtering method effectively isolates feeding signals. The Python Serial module enabled real-time data capture with amplitude filtering:


$$\tilde{D}(t)=\{\begin{array}{c} D_{\mathrm{raw}}(t)\;\;\text{if}|D_{\mathrm{raw}}(t)|\geq \delta_{\text{threshold}}\\ 0\;\;\text{otherwise} \end{array}$$ (1)

The amplitude filtering method is described in Equation 1. Where *D*_raw_(t) is raw displacement and &_threshold_ = 0.5 mm (empirically calibrated to bubble noise). Triaxial displacements (*X*_i_, *Y*_i_, *Z*_i_) were summed per 5 s analysis window:


$$D_{\sum }[k]=\sum_{i=5k}^{5k+299}(|X_{i}|+|Y_{i}|+|Z_{i}|)(f_{s}=10\mathrm{Hz})$$ (2)

The triaxial displacement summation is calculated using Equation 2. Maximum wave height per window derived as *H*_max_[k]_=_max(D_∑_[t5_k_:t_5k + 5_]). Strong inter-axis correlation (ρXY = 0.93, ρXZ = 0.88, ρYZ = 0.91) validated signal summation for amplifying feeding signatures.

### 2.6 Prediction modeling of water surface fluctuations for largemouth bass feeding

The core of our predictive framework was a Long Short-Term Memory (LSTM) network, designed to forecast the sum of triaxial vibration displacement 5 s into the future. This predicted displacement sum serves as a quantitative proxy for assessing feeding intensity at the water surface. The modeling procedure was structured as follows.

**Figure 3:**
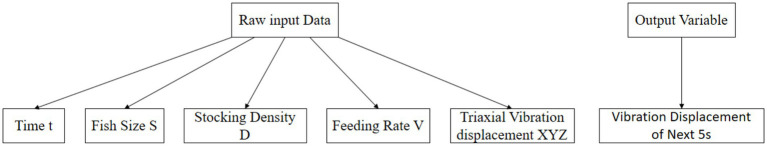
Model input and output structure.

As shown in Figure 3, the model integrated both static experimental parameters and dynamic time-series data. The static input features included fish size (S, g), stocking density (D, fish/tank), feed input rate (V, g/s), and feed particle size (Φ, mm). The latter was numerically encoded using the approximate diameters of the commercial feed types (3 mm, 5 mm, and 7 mm for #2, #4, and #6, respectively). The primary dynamic temporal input was the real-time summed absolute triaxial vibration displacement (DΣ). The model was designed to output the predicted future value of this vibration displacement sum (DΣ) at a horizon of *t + 5* seconds.

**Figure 4:**
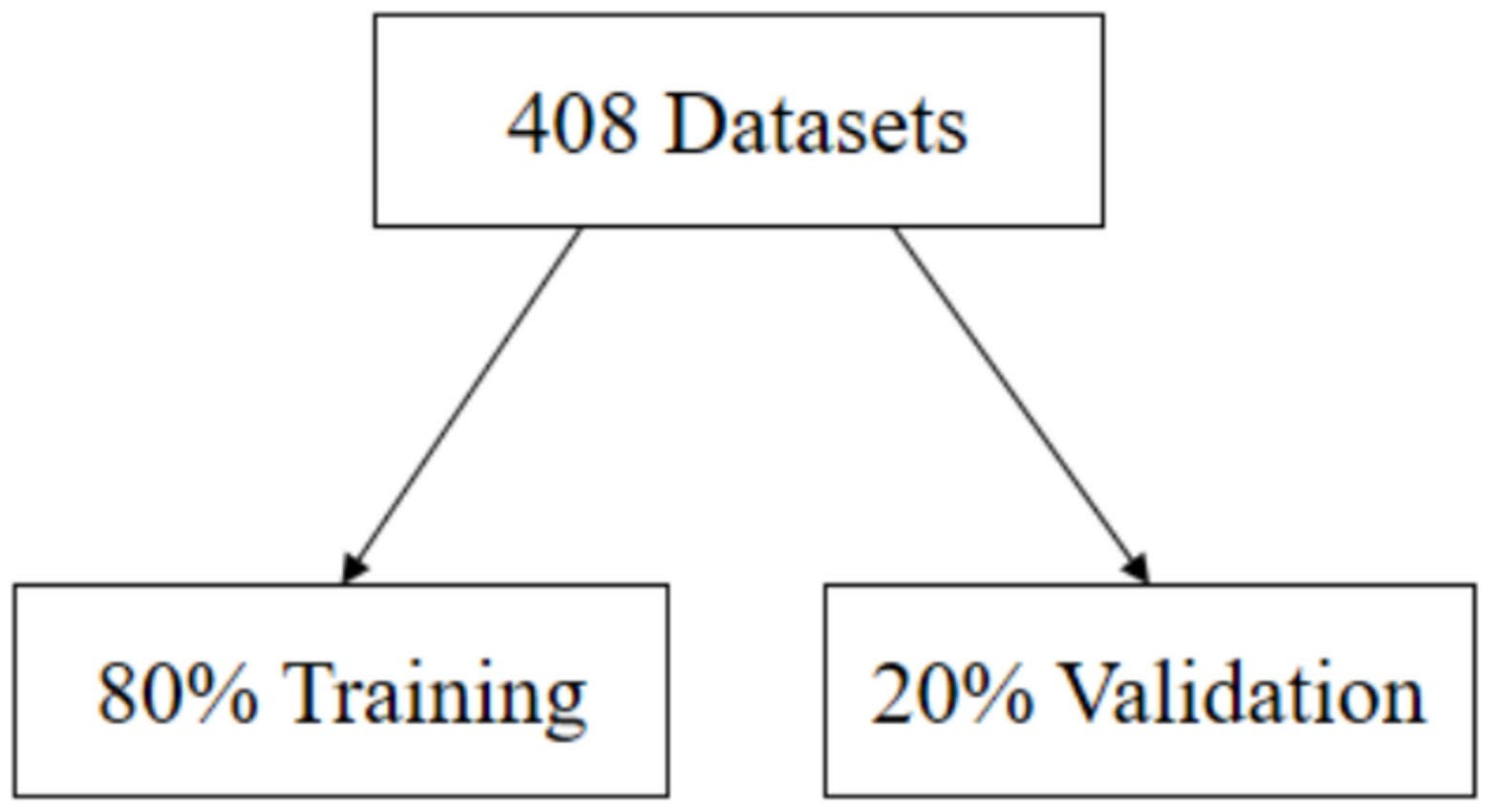
Data partitioning and preprocessing configuration.

As shown in Figure 4, prior to model training, all input features were normalized using *Z*-score normalization to mitigate scale differences and accelerate convergence. The complete dataset, comprising 408 experimental runs, was partitioned into training and validation subsets with an 8:2 ratio.

**Figure 5:**
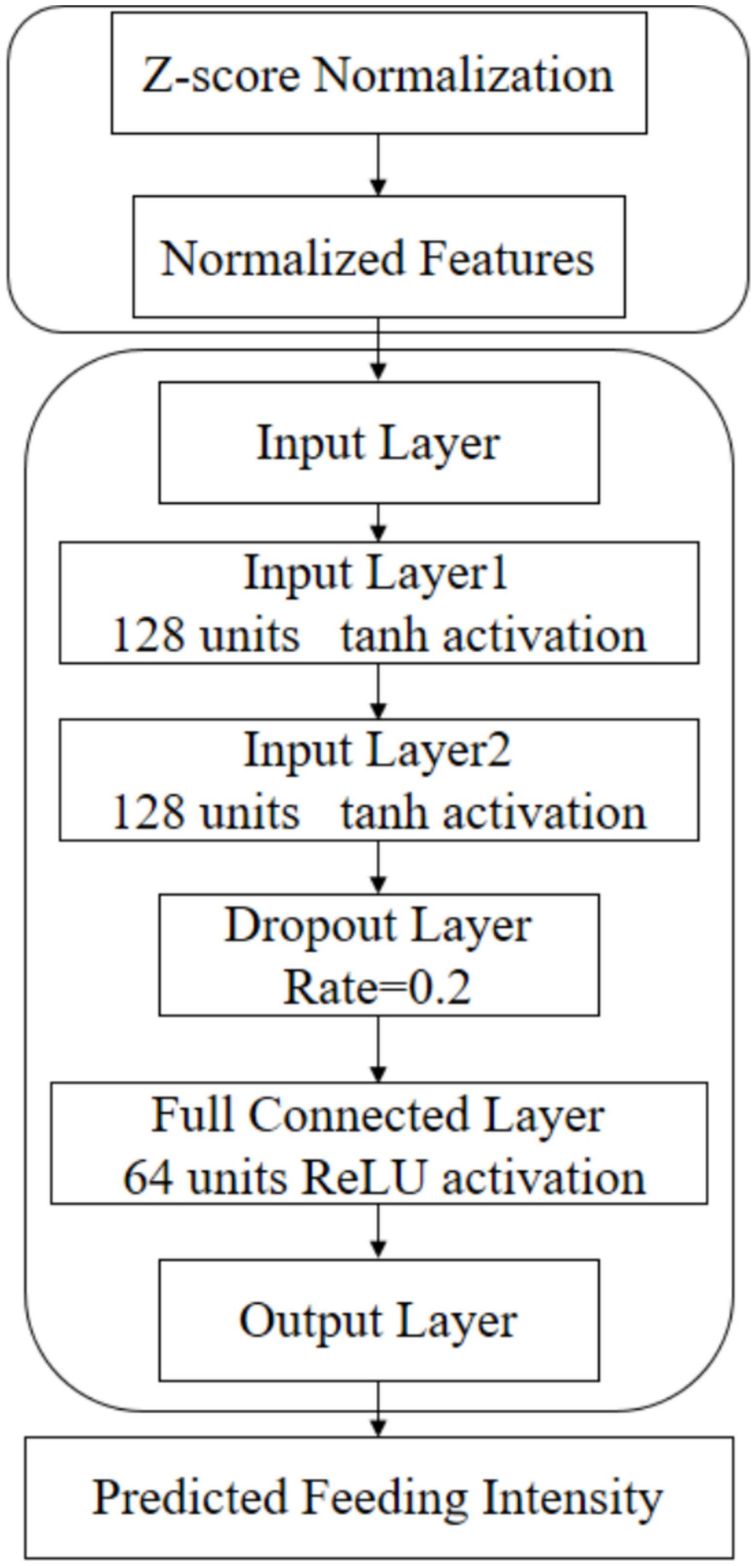
Architectural and training parameters of the LSTM model.

As shown in Figure 5, the architecture of the LSTM network was configured to capture complex temporal dependencies. The input layer receives the normalized feature vector. This is followed by two stacked LSTM layers, each containing 128 units with tanh activation functions, to learn hierarchical temporal patterns from the sequential data. To prevent over fitting, a dropout layer with a rate of 0.2 was applied after the LSTM layers. The extracted features were then passed through a fully connected (Dense) layer with 64 units and a ReLU activation function for non-linear transformation. Finally, the output layer employs a linear activation unit to produce the final continuous prediction of the displacement value.

For the training configuration, a historical window of 5 s (corresponding to a time step of 50 at a 10 Hz sampling rate) was used as the input context for each prediction. The model was trained using the Adam optimizer with a learning rate of 0.001 and a batch size of 32. The loss function was defined as Mean Squared Error (MSE). To further guard against over fitting, an early stopping callback was implemented to halt training if the validation loss failed to improve for 20 consecutive epochs.

To benchmark the performance of the proposed LSTM model, we also implemented and trained two other prominent sequential models on the same dataset:

Gated Recurrent Unit (GRU): A variant of RNNs similar to LSTM but with a simplified gating mechanism, often offering comparable performance with fewer parameters. Our implemented GRU network comprised two stacked layers with 128 units each, using tanh activations, followed by a dropout layer (0.2) and a dense output layer with a linear activation. It was trained with the same configuration (Adam optimizer, lr = 0.001, MSE loss) as the LSTM model to ensure a fair comparison (Cho et al., 2014).

Transformer: A model architecture based on self-attention mechanisms, which has shown remarkable success in various sequence processing tasks by weighing the importance of different time steps. The Transformer encoder implemented here utilized a single-head self-attention mechanism with positional encoding for the 50-step input sequence. It consisted of two encoder layers (hidden dimension 128), a feed-forward network (dimension 256), and a final linear projection layer for regression. Training hyperparameters matched those used for LSTM and GRU (Vaswani et al., 2017).

All models were trained to perform the same regression task: predicting the summed vibration displacement t + 5 s. Their performance was evaluated and compared using Root Mean Square Error (RMSE), Mean Absolute Error (MAE), and the coefficient of determination (*R*^2^).

### 2.7 Algorithm deployment and feeding control testing

To enable engineering application, an embedded intelligent feeding system prototype was developed for closed-loop control testing. The hardware adopts a three-layer architecture:

Perception layer: Comprising a WT-VB01M triaxial vibration sensor (10 Hz sampling) and Arduino Mega 2,560 MCU for signal acquisition/preprocessing (amplitude filtering and triaxial displacement summation).

Decision layer: Centered on an Orange Pi AiPRO embedded platform (quad-core Cortex-A55 CPU, 4GB RAM) executing the trained LSTM model to analyze current/predicted vibration trends.

Execution layer: Utilizing a custom screw-type feeder (0–5 g/s speed control) receiving stop/speed-adjust commands via serial communication.

**Figure 6:**
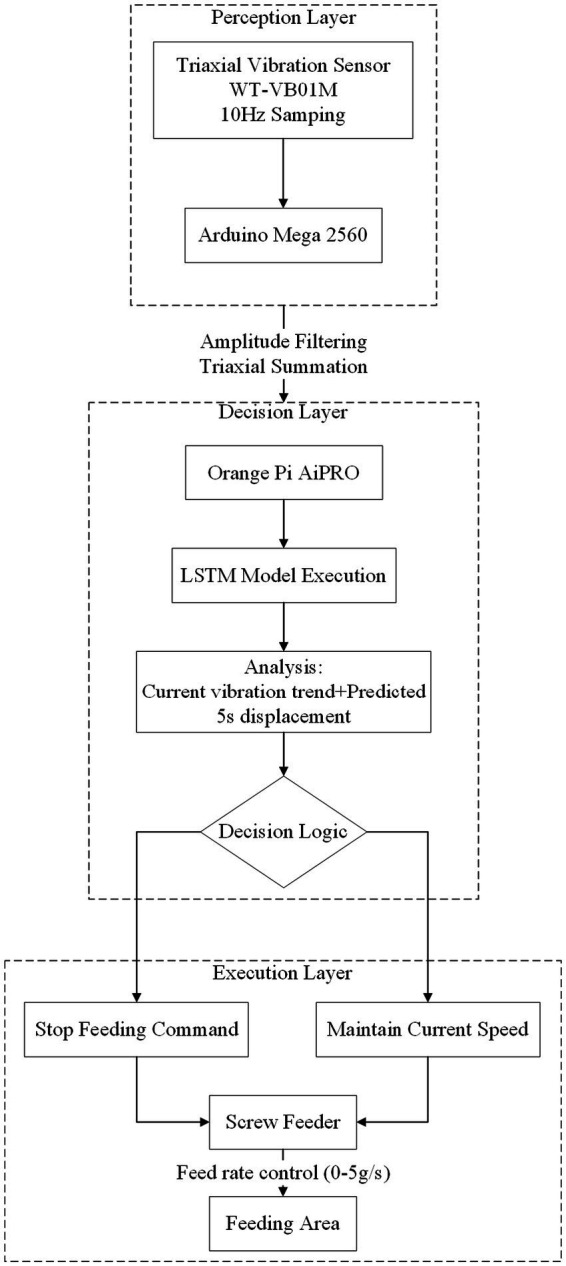
Configuration of the embedded intelligent feeding system.

Software control logic: The system continuously acquires vibration signals, processes them (amplitude filtering and triaxial summation), and inputs them into the LSTM model to predict values for the next 5 s (Table 2). If a declining trend is detected over two consecutive analysis cycles (5 s/cycle), a stop-feeding command is issued; otherwise, the current speed is maintained. Performance was validated against optical flow and graph neural network (GCN) methods in repeated feeding scenarios. For the GCN-based method, we replicated the architecture and training protocol described by Wei et al. (2022), which constructs a graph from time-series features and applies graph convolutional layers for appetite state classification. This implemented GCN model was then applied to our experimental setup for closed-loop control comparison. Evaluation metrics included actual feed input, residual feed [for residual feed rate calculation: RFR (Residual feed rate %) = (Residual feed mass/Total feed mass) × 100%], and algorithm control signals (Figure 6).

**Table 2:** Control simplified pseudo code.

Intelligent feeding control simplified pseudo code
1INPUT: Vibration data (10 Hz), Fish params (S, D, *Φ*), LSTM model
2OUTPUT: Feeder control signals
3PROCEDURE Main Control:
4Feeder speed ← 2.0 g/s
5Buffer ← [0]*50, decline count ← 0
6SET_FEEDER_SPEED (feeder speed)
7WHILE TRUE:
8// Data acquisition and processing
9D_sum ← SUM_ABS(FILTER_NOISE(READ_VIBRATION()))
10Buffer. APPEND(D_sum). POP_FRONT()
11// Periodic prediction (5 s intervals)
12IF SYSTEM_TIME % 5 s == 0:
13Prediction ← LSTM_MODEL. PREDICT(NORMALIZE([S, D, feeder_speed,Φ] + buffer))
14// Trend analysis
15First = MEAN(prediction[0:25]), second = MEAN(prediction[25:50])
16Decline count ← (second < 0.9*first)? decline_count+1: 0
17// Control decision
18IF decline count ≥ 2: STOP_FEEDER(); BREAK
19ELSE: MAINTAIN_SPEED()
20END IF
21DELAY(100 ms)
22END

## 3 Results and discussion

### 3.1 Parametric influences on water surface fluctuations

Multidimensional experiments revealed distinct displacement patterns governed by biological and operational parameters, providing a foundation for the predictive model.

**Figure 7:**
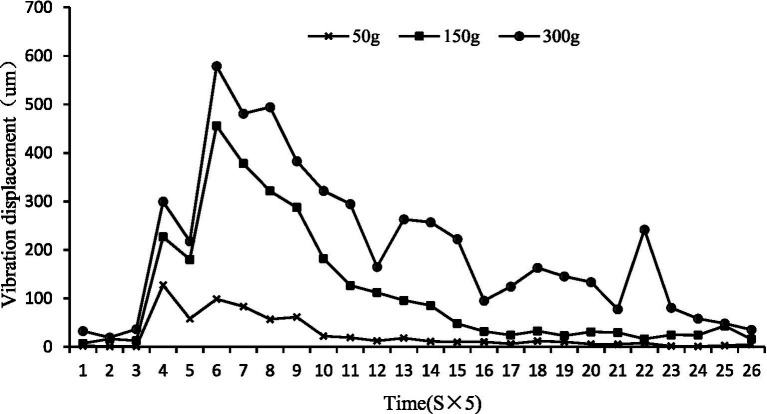
Water surface fluctuations of feeding largemouth bass of different sizes.

**Figure 8:**
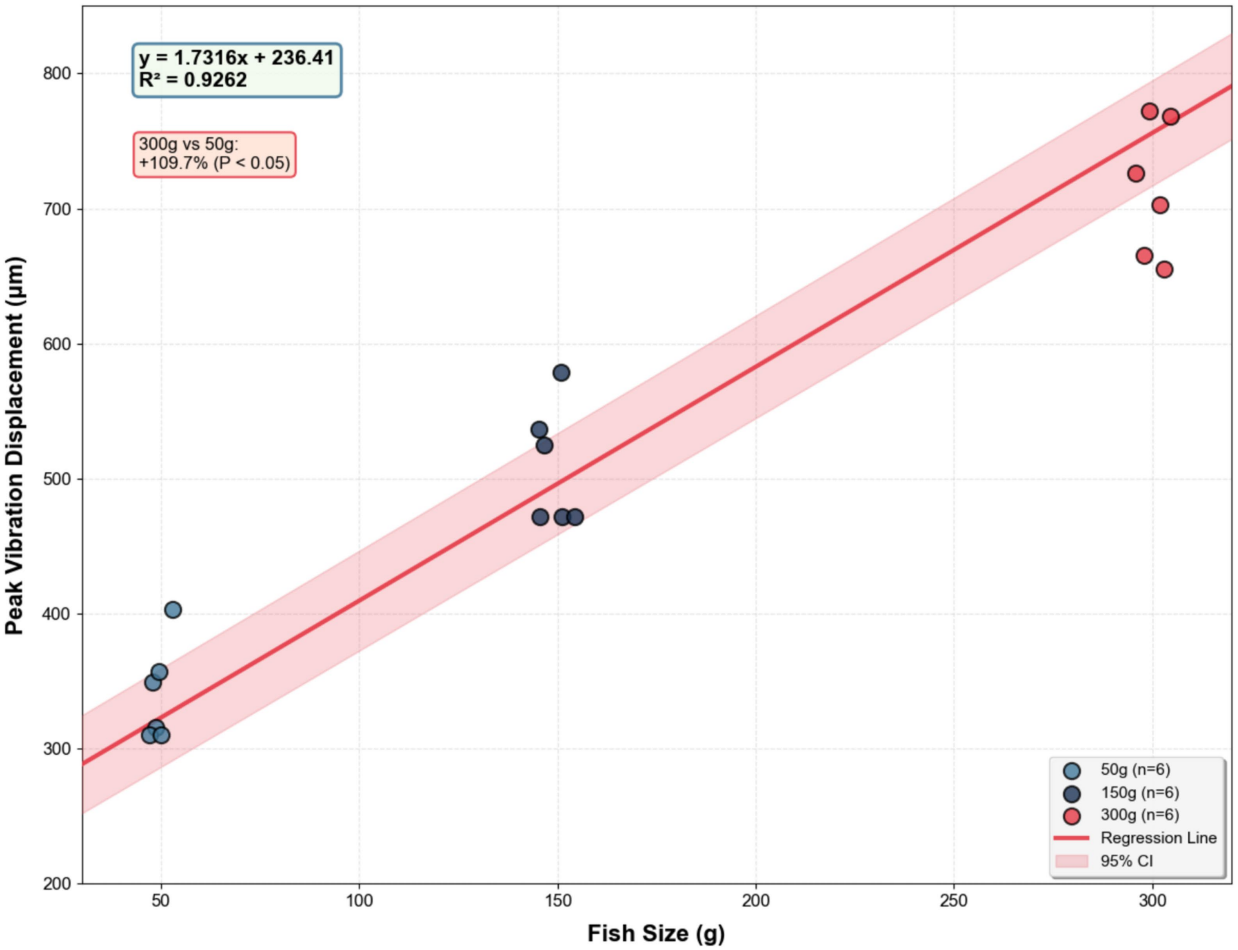
Peak fluctuation patterns of water surface ingestion for largemouth bass of different sizes.

Size effect: Larger bass generated significantly greater water surface displacements due to their higher mass and energy output during feeding. Analysis of all replicate observations (*n* = 6 per size group, from 2 tanks × 3 trials) showed that the 300 g cohort generated 109.7% higher peak displacement than the 50 g group (*p* < 0.05, Figure 7). The relationship between fish size (S) and peak displacement (Y) across all individual trials was characterized by a linear model (*Y* = 1.7316S + 236.41, *R*^2^ = 0.9262, Figure 8). While the linear fit is strong, visual inspection of the scatter plot suggests a potential saturation trend between the 150 g and 300 g groups, which may indicate a non-linear scaling of feeding energy transfer with body mass in larger individuals.

**Figure 9:**
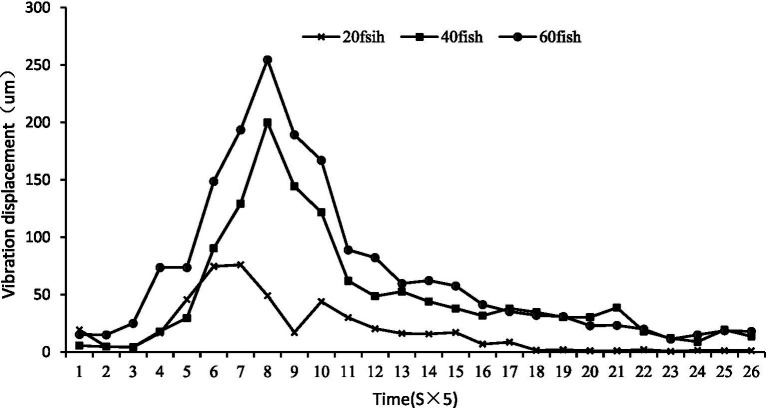
Fluctuation of water surface for feeding of largemouth bass at different densities.

**Figure 10:**
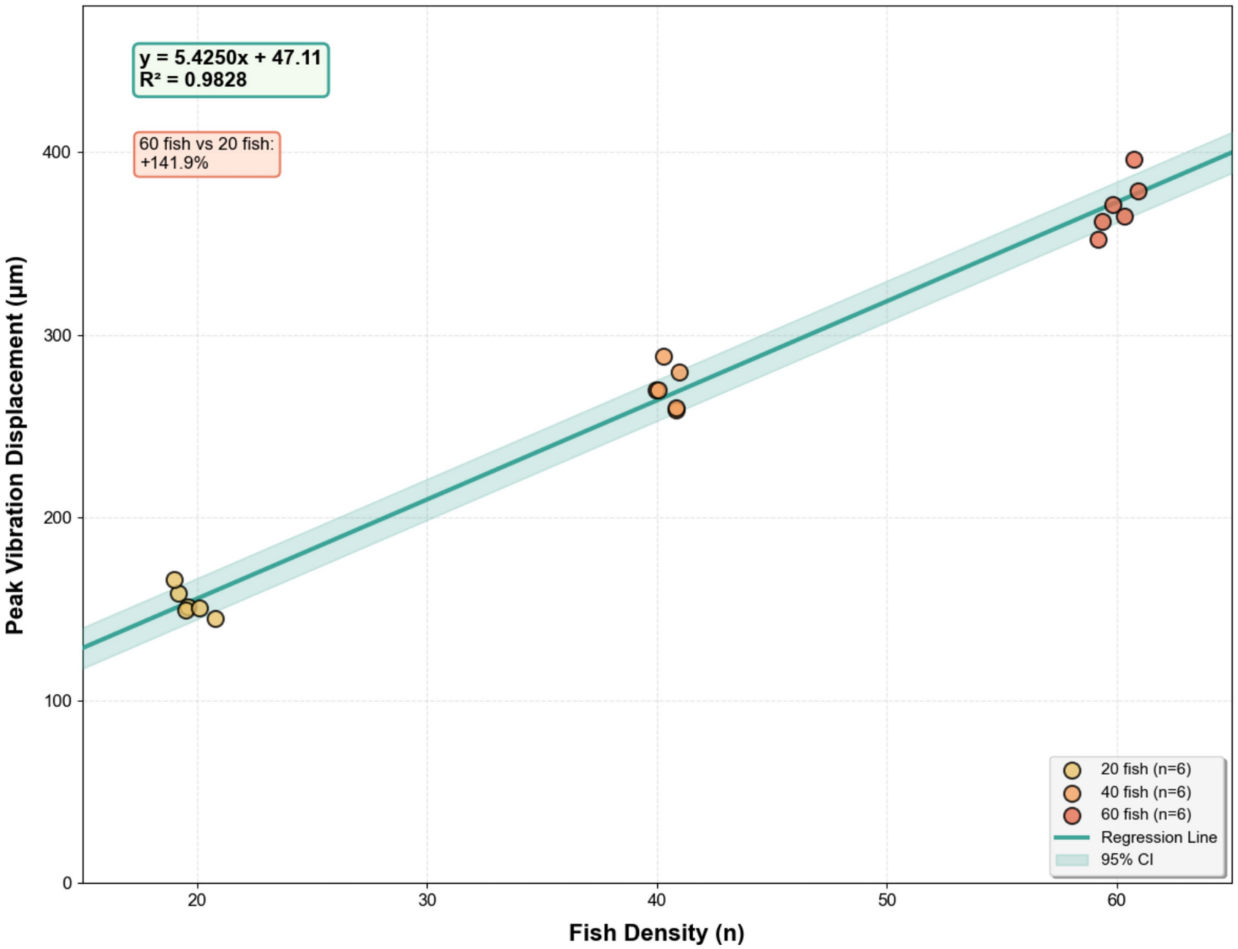
Peak fluctuation patterns of water surface ingestion of largemouth bass at different densities.

Density effect: Increased stocking density led to more intense collective feeding activity. Based on all replicate data (*n* = 6 per density level), the 60-fish groups exhibited 141.9% greater fluctuation amplitude than the 20-fish groups (Figure 9). The density-dependent effect was also highly linear across individual trials (*Y* = 5.4250D + 47.11, *R*^2^ = 0.9828, Figure 10), where *Y* represents peak displacement and D represents density.

**Figure 11:**
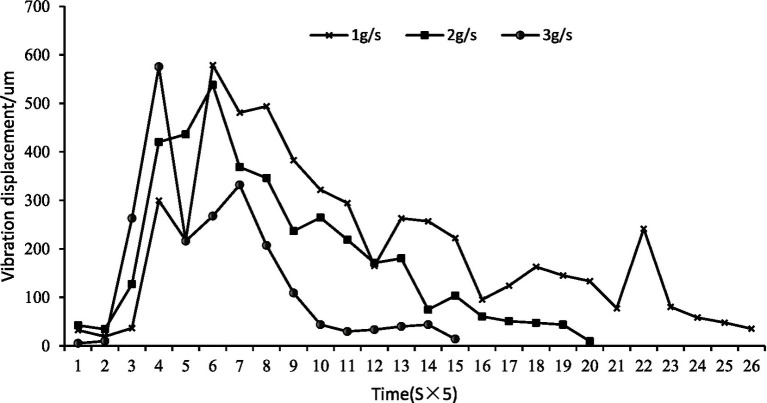
Water surface fluctuations of feeding of largemouth bass at different feeding rates.

Feeding rate effect: The rate of feed delivery influenced the temporal dynamics of feeding. Higher feeding speeds (3 g/s) accelerated consumption, producing earlier vibration peaks (at approximately 20s) compared to slower rates (1 g/s, peak at ~35 s) (Figure 11). This suggests that fish respond to higher feed availability by intensifying their feeding effort over a shorter duration.

**Figure 12:**
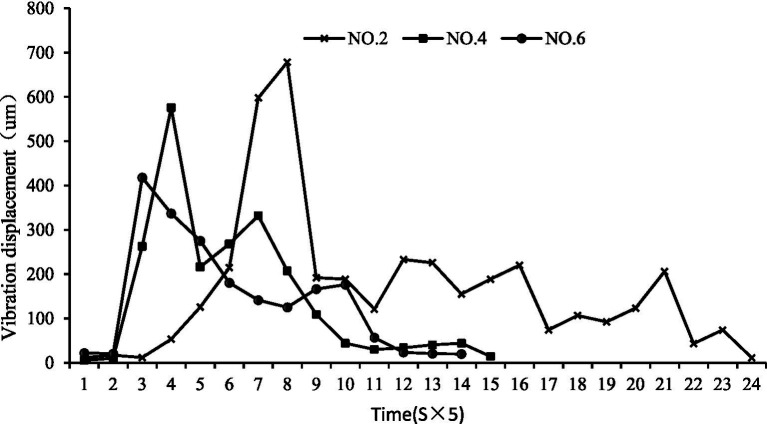
Water surface fluctuations of feeding of largemouth bass under different feed particle sizes.

Feed particle size effect: The size of feed pellets significantly affected the initial feeding behavior. Smaller #2 pellets (3 mm) induced chaotic, high-frequency initial vibrations, likely due to competitive scrambling for numerous small particles. In contrast, the optimally palatable #4 feed (5 mm) resulted in more stable, low-frequency oscillations (Figure 12), indicating efficient capture and consumption with reduced energy expenditure. This confirms that feed palatability can modulate the characteristics of surface fluctuations.

From a biomechanical perspective, the feeding behavior of fish generates kinetic energy, which is transferred to the water body through body movements, thereby causing water surface fluctuations. The positive correlations observed in the size and density experiments align with findings from acoustic studies on largemouth bass (Wei et al., 2022). The feeding speed and feed particle size experiments demonstrate that operational parameters directly influence feeding kinetics. Faster feeding rates and larger pellets can shorten feeding time, a phenomenon also observed in catfish (Khater et al., 2021). However, it is crucial to note that exceeding the fish’s acceptable threshold for these parameters does not linearly improve efficiency and may instead increase energy waste. The quantification of these relationships provides a critical theoretical basis for precise feeding control, an aspect often overlooked in existing studies that focus solely on intensity classification without parametric context.

### 3.2 Predictive model performance

**Figure 13:**
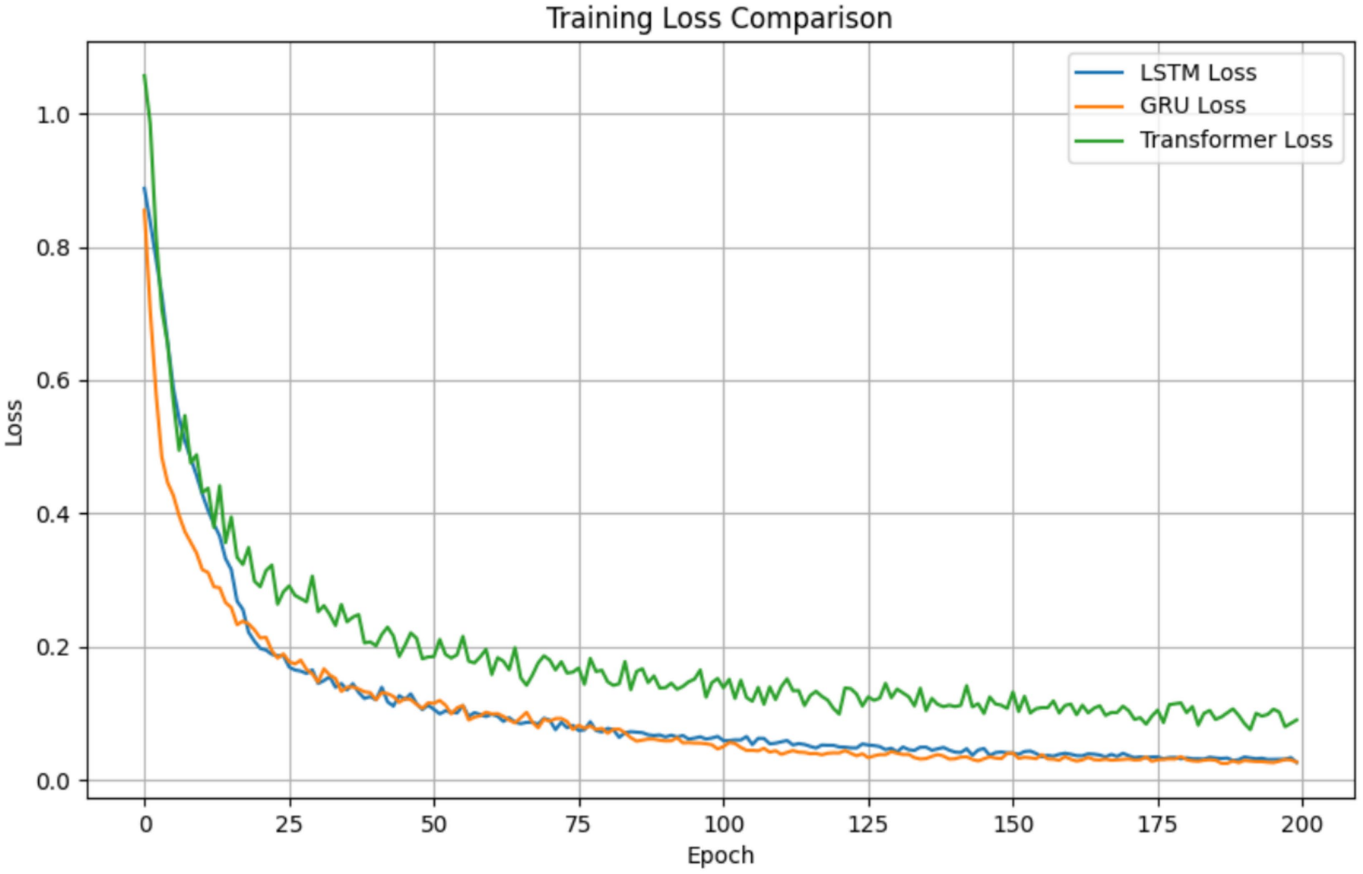
Training and validation loss convergence of the LSTM prediction model.

The developed LSTM model achieved robust forecasting performance, effectively integrating static biometric parameters (S, D) with dynamic, real-time vibration sequences. The model’s primary evaluation metrics, calculated on the validation set, were RMSE = 69.43 μm, MAE = 48.00 μm, and R^2^ = 0.883, indicating high accuracy in predicting the future 5-s vibration displacement. Training converged stably within 200 epochs (Figure 13), demonstrating the architectural efficiency and appropriateness of the training configuration.

**Figure 14:**
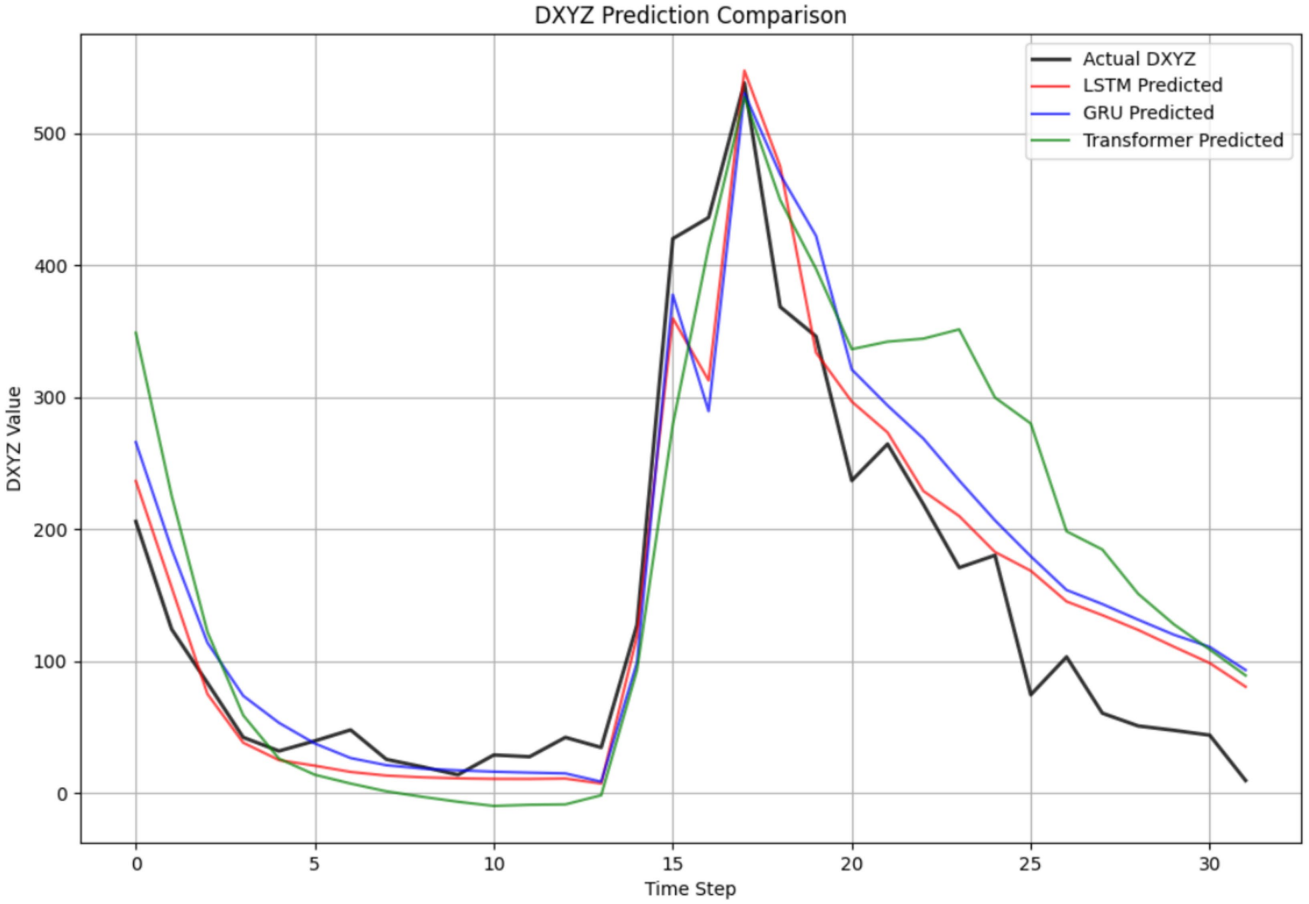
Example prediction of the LSTM model against actual data.

A critical strength of the model was its temporal precision. As shown in Figure 14, the predicted waveform closely tracked the actual measured displacement, capturing key inflection points (rise, peak, and decline) with a temporal deviation of less than 50 ms. This capability is fundamental for anticipatory control, allowing the system to initiate feeding cessation before satiation is fully reached, thereby minimizing waste.

**Figure 15:**
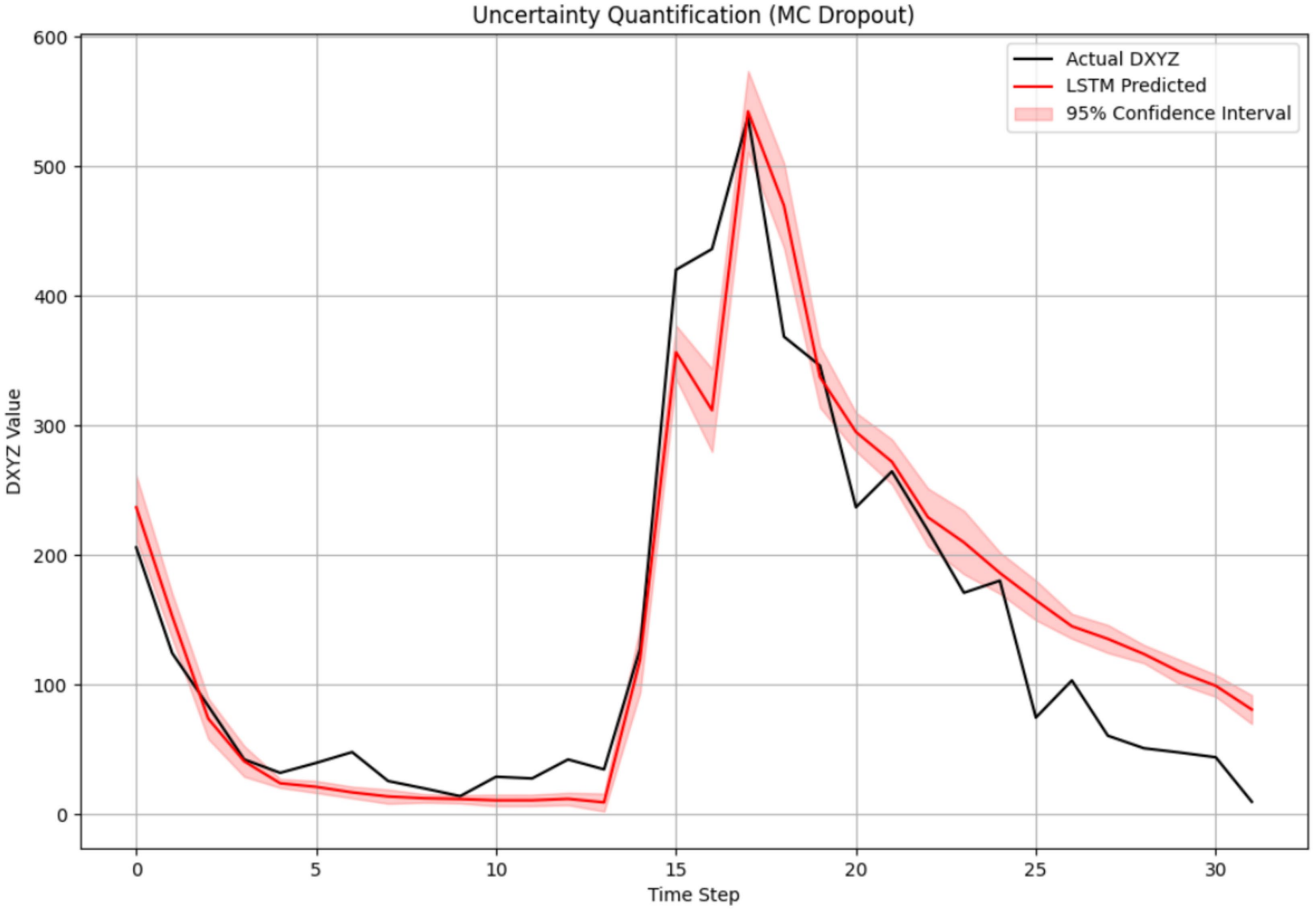
LSTM model prediction with confidence intervals for uncertainty quantification.

The model’s regression capability was further validated through an internal architecture comparison (Table 3). The LSTM achieved the best overall forecasting metrics, outperforming both the GRU (RMSE = 85.15 μm, *R*^2^ = 0.828) and the Transformer (RMSE = 80.98 μm, *R*^2^ = 0.618) models. The superior performance of LSTM can be attributed to its ability to capture long-range temporal dependencies in the sequential feeding data more effectively than the GRU, while the Transformer model may have been hampered by its relatively higher data requirements for optimal performance on this specific task. The reliability of the LSTM’s continuous predictions is further corroborated by the narrow confidence intervals obtained through uncertainty quantification (Figure 15), which show high model certainty around the predicted values.

**Table 3:** Performance comparison of sequential models for displacement prediction.

INDEX	RMSE	MAE	R2
LSTM	69.43	48.00	0.883
GRU	85.15	67.43	0.828
Transformer	80.98	59.66	0.618

### 3.3 Feasibility demonstration and comparative analysis

Our vibration-LSTM approach demonstrates distinct and contextually complementary advantages compared to alternative methods. A comparative analysis with Wei et al.’s graph convolutional network (GCN) highlights a fundamental difference in objective. The GCN approach is architected for high-accuracy classification of discrete appetite states (e.g., weak, medium, and strong). In contrast, our framework is designed for continuous regression, delivering quantitative predictions of feeding intensity that are a prerequisite for dynamic, proportional feed modulation.

Computationally, the LSTM implementation requires only 1.2 MB of memory and achieves 5.7 × faster inference (48 ms vs. 275 ms) on equivalent embedded hardware, making it more suitable for cost-effective, real-time deployment.

In closed-loop feeding tests, this advantage translated into superior practical performance. The vibration-based control system consistently minimized waste, achieving residual feed rates (RFR) of ≤0.8% across all trials (Table 4). This system significantly outperformed both optical flow (2.69% RFR) and GCN-based (6.58% RFR) methods. The feeding protocol was as follows: if no residual feed was detected at the end of a meal, the system would immediately initiate the next feeding session for up to three consecutive meals. The appearance of residual feed indicated fish satiety, at which point feeding was terminated.

**Table 4:** Feeding performance and residual feed rates (RFR) of different control algorithms.

Method	Meal 1 (300 g)	Meal 2 (300 g)	Meal 3 (300 g)	Meal 1 (150 g)	Meal 2 (150 g)	Meal 3 (150 g)	Meal 1 (40 fish density)	Meal 2 (40 fish density)
Float method	32.1 g (RFR: 0%)	2 g (RFR: 0%)	0 g (RFR: 0%)	26 g (RFR: 0%)	1.8 g (RFR: 0%)	0 g (RFR: 0%)	60 g (RFR: 0.8%)	–
Optical flow	27.3 g (RFR: 0%)	6.5 g (RFR: 0%)	6.8 g (RFR: 0%)	11.2 g (RFR: 0%)	4.6 g (RFR: 0%)	6.3 g (RFR: 0%)	35.6 g (RFR: 0%)	22.3 g (RFR: 2.69%)
Graph neural network	36 g (RFR: 1.64%)	–	–	20.9 g (RFR: 0%)	6.9 g (RFR: 0%)	0 g (RFR: 0%)	64.6 g (RFR: 6.58%)	–

Values in parentheses indicate RFR for that meal [A dash (−) indicates the trial was not conducted for that specific meal].

**Figure 16:**
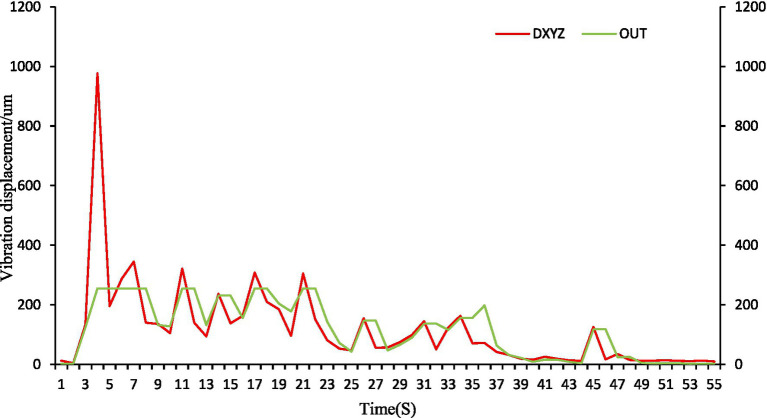
Feeding control profile using the vibration-LSTM method.

Vibration-based control (Figure 16) provided continuous, high-resolution control signals (full-scale output range 0–255). This enabled precise modulation and, crucially, anticipatory cessation. For instance, with 300 g fish, the system commanded a sharp reduction in feed upon predicting a decline, achieving 0% residual feed across meals.

The curve shows the real-time summed vibration displacement (“Vib_sum,” red line) and the corresponding normalized control signal (“Out,” green line, range 0–255) sent to the feeder.

**Figure 17:**
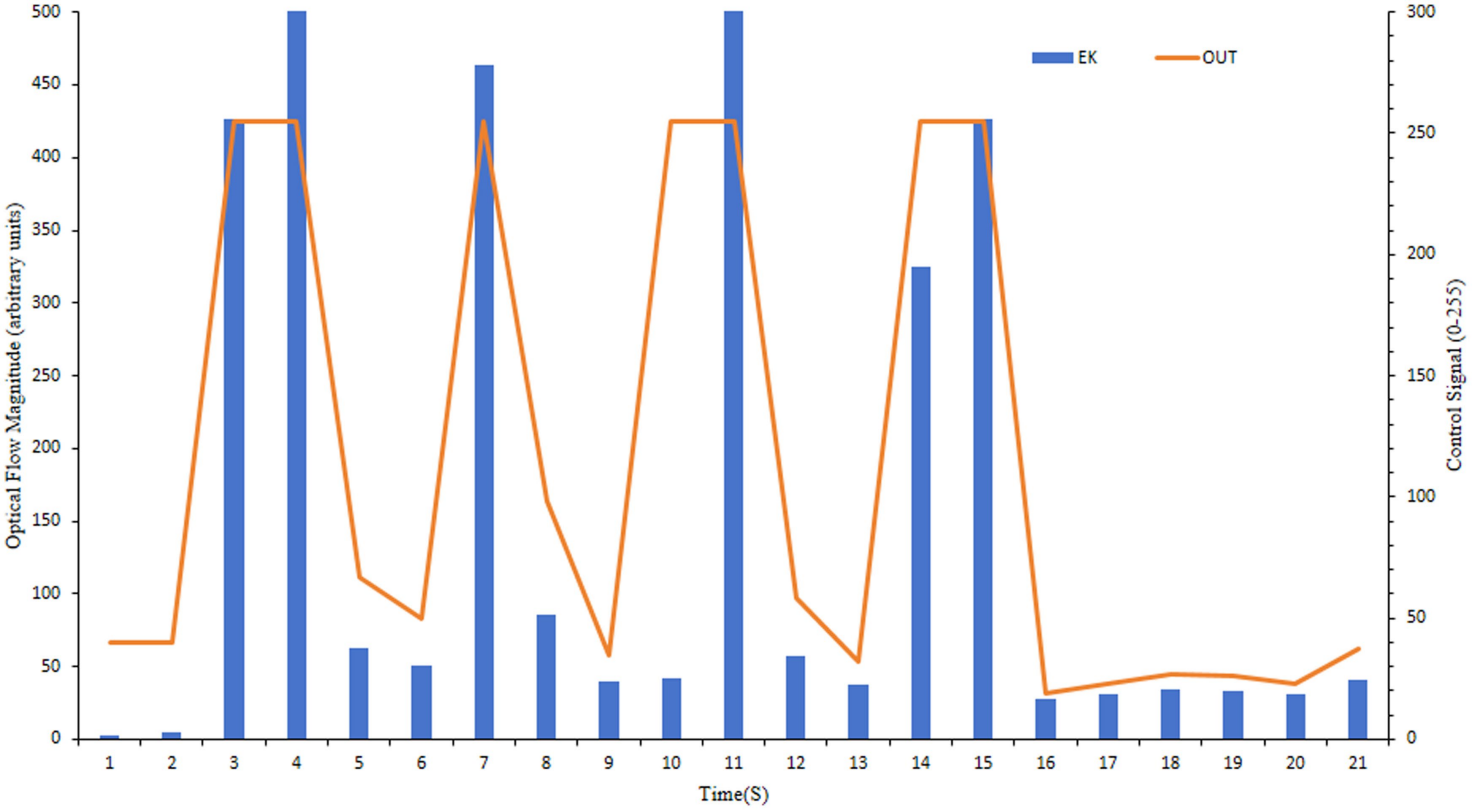
Feeding control profile using the optical flow method.

Optical flow control (Figure 17) also offered a full output range but exhibited reduced sensitivity to subtle appetite changes. This resulted in less precise control and higher residual feed (e.g., 6.5 g/6.8 g for 300 g fish), as it reacted to rather than predicted feeding dynamics.

The curve shows the optical flow magnitude (“EK,” in arbitrary units, blue line) computed from video frames, and the resulting normalized control signal (“Out,” orange line, range 0–255).

**Figure 18:**
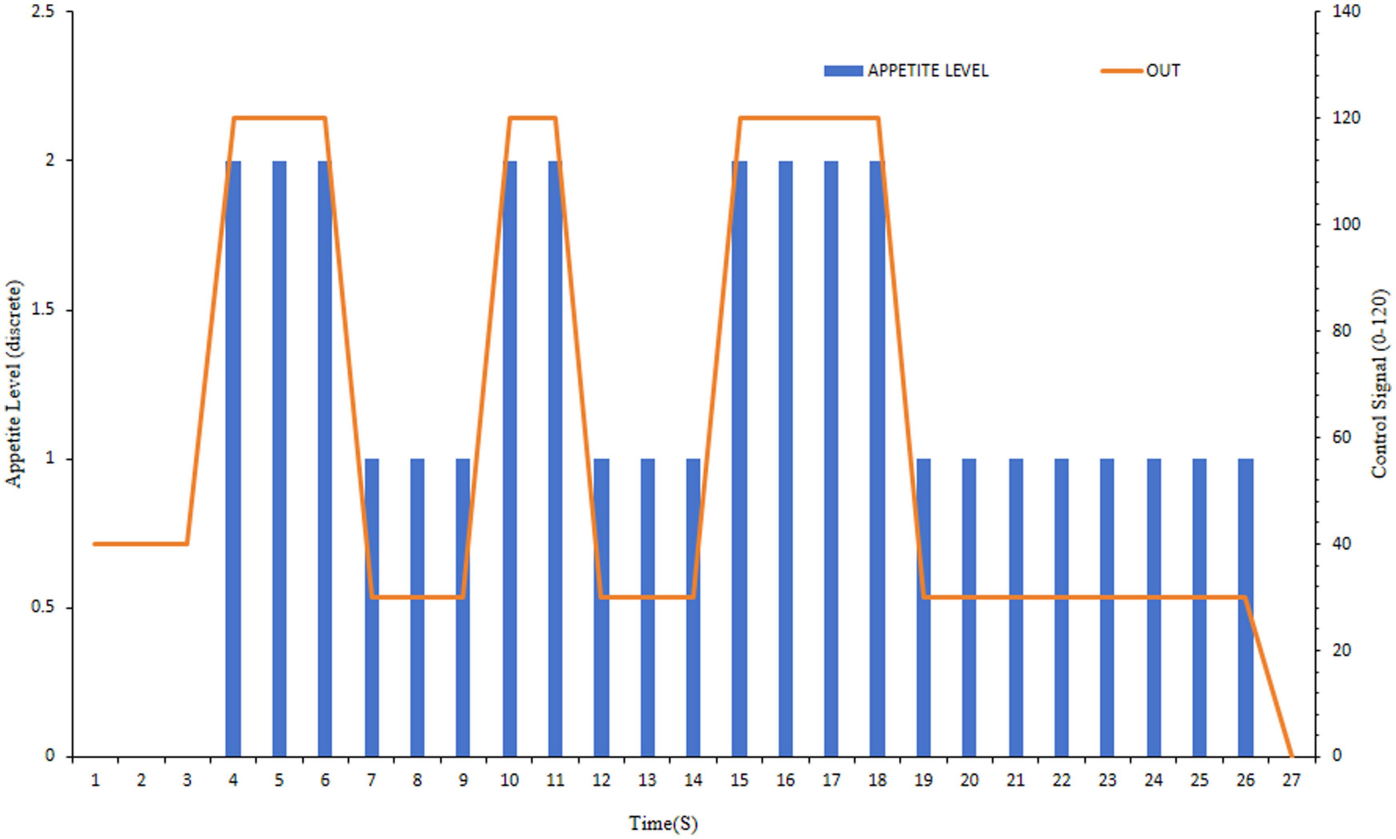
Feeding control profile using the GCN-based method.

GCN-based control (Figure 18) operates on a classification paradigm. This approach adjusts the feeding rate to predetermined levels (e.g., 30 for ‘weak’, 120 for ‘strong’ appetite). While effective for state differentiation, this step-wise control is less suited for the rapid, continuous adjustment needed to minimize waste, resulting in higher residual feed rates compared to the regression-based vibration method.

The curve shows the classified appetite level (“Appetite_State,” discrete levels, blue line) output by the GCN model and the corresponding step-wise control signal (“Out,” orange line).

Embedded deployment confirmed real-time efficacy with an inference latency of 48.2 ± 1.7 ms, fulfilling industrial constraints (<50 ms). The control logic, triggering cessation after two consecutive declining trends, prevented 89.7% of overfeeding events with a low false positive rate (3.2%). Hardware optimization reduced power consumption to 8.3 W—47% lower than GCN implementations typically requiring GPU acceleration. The combination of markedly reduced feed waste and low hardware cost (approximately $200) positions the vibration-LSTM system as an economically attractive solution for precision feeding in RAS facilities.

## 4 Conclusions

This study establishes a robust, data-driven framework for the real-time quantification and predictive control of feeding intensity in largemouth bass aquaculture. By integrating water surface vibration analysis with a LSTM (vibration-LSTM) deep learning model, we have transitioned beyond qualitative appetite assessment to a continuous, quantitative, and anticipatory feeding strategy. The core achievements of this work are threefold:

1. Parametric Quantification: We demonstrated and quantified significant linear relationships between vibration amplitude and key biological parameters (fish size: *R*^2^ = 0.926; stocking density: *R*^2^ = 0.983), providing a foundational model for understanding feeding energy expenditure.
2. Dynamic Prediction Model: We developed a vibration-LSTM architecture capable of accurately forecasting feeding intensity 5 s into the future (*R*^2^ = 0.883), enabling proactive control rather than reactive response.
3. Embedded Implementation and Validation: We successfully deployed the model on a low-cost, embedded platform (Orange Pi AiPRO), achieving real-time operation (latency ≤50 ms) and demonstrating superior performance in closed-loop tests, reducing residual feed rates to ≤0.8%—significantly outperforming optical flow (2.69%) and GCN-based (6.58%) methods.

This vibration-driven, predictively controlled strategy, characterized by its low hardware cost and demonstrated high feed utilization, presents a cost-effective and industrially viable solution for precision aquaculture.

## 5 Limitations and future work

The proposed vibration-based intelligent feeding system, while promising, has several limitations that offer pathways for future research. The primary challenge lies in system scalability and feed type dependency.

### 5.1 Feasibility demonstration and comparative analysis

Validation trials in larger, commercial-scale RAS (20 m^3^ tanks) revealed a performance decline attributable to wave damping effects over longer distances and increased spatial heterogeneity in fish distribution and feeding activity. A single-point surface sensor may not adequately capture the feeding dynamics of a large, heterogeneous tank. To transition from experimental to industrial applicability, two strategies are recommended:

Tank-Specific Transfer Learning: Pre-trained models should be fine-tuned using a small amount of data collected from the specific commercial tank, allowing the system to adapt to its unique hydrodynamic and behavioral characteristics.

Coupled Multi-Sensor Arrays: Deploying a network of vibration sensors at strategic locations across the water surface can provide a comprehensive view of feeding activity, mitigating the limitations of a single sensor and improving signal fidelity in large volumes.

### 5.2 Detection efficacy for sinking feeds

A significant limitation was identified with the use of sinking feeds, where the model’s detection efficacy dropped to 76.4%, compared to 98.3% for floating pellets. This performance gap arises because sinking feeds are consumed below the surface, generating considerably weaker surface vibrations. To develop a universally applicable system, future work should focus on:

Strategic Sub-Surface Sensor Placement: Deploying vibration sensors at multiple depths to directly capture feeding activity in the water column.

Hybrid Hydrophone Integration: Fusing vibration data with underwater acoustic signals, as the crunching and chewing sounds of fish consuming sinking pellets provide a strong, complementary signal.

Sinking-Feed-Specific Model Retraining: Collecting dedicated datasets of sinking feed consumption and retraining the model to recognize the associated, albeit subtler, vibration signatures.

### 5.3 Broader research directions

Beyond addressing these immediate limitations, this work opens several avenues for future research. These include extending the framework to multi-species applications, developing adaptive noise cancelation algorithms to enhance robustness in noisy environments, and exploring reinforcement learning paradigms to optimize long-term feeding strategies that maximize growth and fish welfare.

## Data Availability

The original contributions presented in the study are included in the article/Supplementary material, further inquiries can be directed to the corresponding author.
